# Clinical Evidence: Metastases can Metastasize

**DOI:** 10.4021/wjon406w

**Published:** 2012-07-05

**Authors:** Gregory A. Stanley, Jyoti P. Balani, David S. Miller, John C. Mansour, Roderich E. Schwarz

**Affiliations:** a Division of Surgical Oncology and Department of Surgery, Simmons Comprehensive Cancer Center, University of Texas Southwestern Medical Center, Dallas, TX, USA; b Department of Pathology, University of Texas Southwestern Medical Center, Dallas, TX, USA; c Department of Obstetrics and Gynecology, Simmons Comprehensive Cancer Center, University of Texas Southwestern Medical Center, Dallas, TX, USA

**Keywords:** Ovarian cancer, Metastasis, Monotherapy, Vinorelbine, Papillary serous carcinoma

## Abstract

We report the unusual case of a 52-year-old female with known stable metastatic ovarian cancer presenting with a new, rapidly growing gastric metastasis, leading to surgical resection. Histologic assessment of the specimen revealed evidence of submucosal and intramuscular metastatic disease originating from a metastatic lesion and not from the primary tumor. This case represents one of an otherwise rarely documented clinical scenario that a metastatic focus can itself metastasize.

## Introduction

The patient is an otherwise healthy 52-year-old female that presented for evaluation of liver-directed therapy in July 2010. She was initially diagnosed at age 36 with Stage IB serous ovarian cancer of low malignant potential in 1994. At that time, she underwent a total abdominal hysterectomy with bilateral salpingo-oophorectomy, and was subsequently treated with adjuvant chemotherapy.

**Figure 1:**
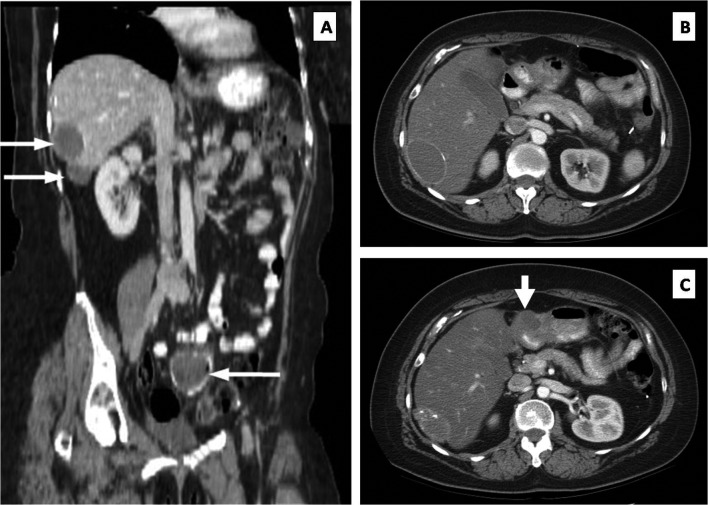
(A) Stable liver metastases (upper white arrows) and pelvic metastasis (lower arrow). (B) Axial CT showing no obvious peritoneal mass at the pylorus. (C) Repeat axial CT performed one year later documenting a new mass (thick white arrow) at the level of the pylorus.

In 1997, the patient underwent pelvic tumor debulking and excision of multiple invasive peritoneal implants for recurrence. At the completion of the operation, no gross evidence of disease was seen. In the years following, she was treated with a variety of chemotherapeutic regimens including agents such as altretamine, etoposide, and gemcitabine, which were met with a variety of ultimately limiting side effects. She finally started treatment with vinorelbine (Navelbine) in April 2001 and tolerated this medication well. Surveillance computed tomography (CT) in 2003 once again revealed evidence of pelvic recurrence in addition to two surface liver masses consistent with peritoneal implants, which are seen in the coronal CT cuts shown in Figure 1A (thin white arrows). Close clinical and radiologic follow-up demonstrated a stable appearance of the pelvic mass and no change of either liver mass upon approaching her 94th cycle of vinorelbine in February 2009. Her CA-125 level ranged from 34.3 to 86.7 U/mL during this period of latency.

One month later, the patient presented with lower abdominal pain and CT evidence of extraluminal air within the pelvic tumor consistent with super-infection from an adjacent rectosigmoid perforation. She underwent a sigmoid colectomy with additional cytoreduction and completion omentectomy. The liver surface implants were confirmed, and a small peritoneal implant was identified overlying the anterior surface of the gastric antrum; these upper abdominal lesions were to be resected in a staged procedure. Upper abdominal cuts of the pre-operative CT scan at the level of the pylorus did not depict this lesion (Fig. 1B). Histolopathologic examination showed metastatic low grade papillary serous ovarian carcinoma with micropapillary features. She recovered without incident and resumed vinorelbine post-operatively, but did not agree to the staged operation at this time.

By mid-2010, an abdominal CT showed asymptomatic enlargement of the stomach implant located on the anterior surface of the pre-pyloric gastric antrum, now measuring 4-centimeters in diameter (Fig. 1C; thick white arrow). The liver masses remained unchanged. The patient now agreed with resection of all residual disease. The liver surface lesions were resected first, followed by a distal gastrectomy, proximal duodenectomy and loop gastrojejunostomy (Billroth II procedure) with regional lymphadenectomy, including a noticeably enlarged lymph node overlying the proper hepatic artery. Postoperatively, the patient had an uneventful hospital course and was discharged after eight days.

**Figure 2:**
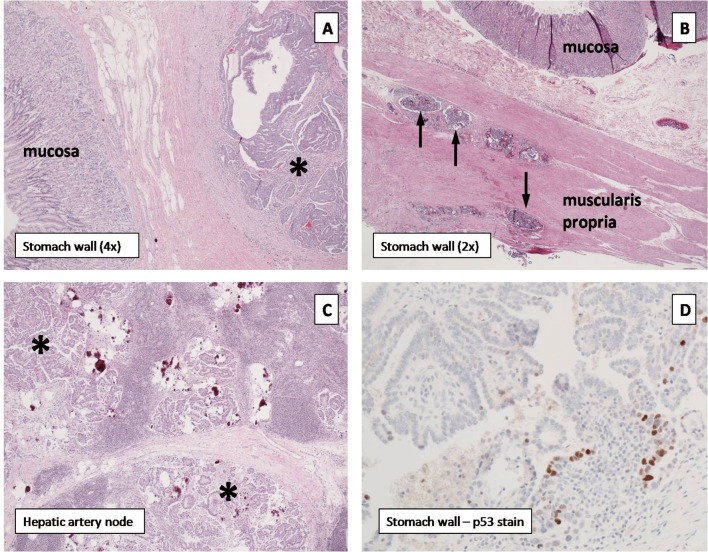
(A) Pyloric tumor (asterisk) invading from serosa without mucosal involvement. (B) Tumor cells within the subserosal, intramuscular, and submucosal lymphovascular channels (black arrows). (C) Hepatic artery lymph node containing metastatic foci (asterisks). (D) P53 immunohistochemical staining confirms low-grade papillary serous carcinoma of the ovary.

Histopathologic analysis confirmed the distal gastric implant to be metastatic low grade papillary serous carcinoma with solid and cystic components. Notably, histologic evaluation revealed this lesion to be invading from the perigastric adipose tissue through the gastric wall into the submucosa without mucosal involvement (Fig. 2A, asterisk denotes tumor). Widespread tumor emboli were seen in the subserosal, intramuscular, and submucosal lymphovascular channels (Fig. 2B, thin arrows). The proximal and distal specimen margins were positive for tumor emboli in the lymphovascular channels. The hepatic artery lymph node contained a 1.6-centimeter focus of metastatic disease with extranodal extension (Fig. 2C, asterisks denote tumor). P53 immunohistochemical staining of all sites of tumor was consistent with the diagnosis of low-grade papillary serous carcinoma of the ovary (Fig. 2D). The patient is currently doing well without complaints. She continues on a vinorelbine regimen with close follow-up and surveillance.

## Discussion

This patient represents one of otherwise rarely documented clinical cases with evidence that a metastatic focus can itself metastasize through a process that does not parallel the mechanism of previous metastasis. The details of this case suggest that a metastatic implant of ovarian carcinoma seeded the serosal tissue overlying the anterior gastric wall. This invasive lesion then eroded through the layers of the stomach from the serosa inward and infiltrated the lymphovascular channels at multiple levels. Further progression then proceeded via these common metastatic pathways to establish a colony of tumor cells within a regional lymph node. As such, the gastric implant assumed the role of a primary cancer, an unexpectedly remarkable event.

While many have speculated that this phenomenon is theoretically possible, to date only a limited number of experimental efforts have been reported to substantiate this assertion. In 1976, Fidler and Nicolson published one of the earliest studies demonstrating the ability of lung metastases to spread to a distant cancer-free site in a murine model, thus creating tertiary metastases [1]. This was accomplished by injecting malignant melanoma cells into the leg of syngeneic mice and documenting lung metastases four weeks later. The leg containing the primary tumor was amputated, and the diseased mice were then parabiotically connected to healthy cancer-free mice. After two weeks of parabiosis, the mice were surgically separated. Thereafter, 40% of the “healthy” mice developed lung metastases, proving that lung metastases can indeed produce additional metastases.

It is clear from this study and the current case that metastatic tumor cells can retain the genetic framework to repeat the metastatic sequence. Nevertheless, how prevalent these mechanisms for secondary metastasis are in human disease remains unknown. The cell lines used in the Fidler study were predisposed to metastasize to the lung through a hematogenous route, and this same mechanism was subsequently observed in order to establish tertiary metastases in the healthy animal; however, prolonged circulation of free tumor cells originating in the primary tumor, or stem cells from a different site than the established metastases that can give rise to new metastases have not been conclusively ruled out. Interestingly, our current case demonstrated transperitoneal migration and implantation followed by predominantly lymphatic invasion and embolization, thus exhibiting a second and obviously alternate mode of metastasis. Nonetheless, observation of this process in a spontaneously occurring human malignancy confirms the clinical presence of a mechanism initially demonstrated by Fidler and Nicolson 35 years ago.

Why did the gastric implant behave much more aggressively than the liver lesions, which were documented to have remained stable for many years? One explanation may be the impact of long-term monotherapy with vinorelbine, contributing to genetic selection and drug resistance. Chronic systemic chemotherapy may lead to selective or adaptive genetic alterations of the tumor cells that can confer drug resistance [2], a mechanism that has been documented for instance to occur in lung cancer [3] and gastrointestinal stromal tumors [4]. While the liver surface implants clearly responded to vinorelbine, the gastric wall implant may have facilitated mutations that allowed development of a clonal population resistant to therapy. Vinorelbine is commonly used for ovarian cancer and has been shown to be susceptible to tumor resistance through a P-glycoprotein-mediated mechanism [5].

Another consideration is the contemporary idea known as *metastatic speciation*, which seems a likely mechanism at work in this case. This theory proposes that as disseminated tumor cells invade a variety of distant organs through metastatic means, the unique selective pressures of each microenvironment alters the ability of the tumor cells to establish a metastatic colony [6]. This usually occurs during a period of cell latency, which can last from months to years. The current case may represent an excellent example of this concept. Once the invasive peritoneal implant infiltrated the gastric wall, local microenvironmental factors may have stimulated aggressive invasion and further tumor colonization. Without the same stimulation by surrounding tissues, such as in the liver, tumor cells may simply remain dormant, behaving in a relatively indolent manner. Alternatively, similar local factors may have inherently selected out a more aggressive tumor cell clone that thrived in the gastric tissue; this is not likely however, as we have to assume that the gastric implant was likely present at the time of the first treatment and it took sixteen years for this process to gain traction. Therefore, it is also possible that the specific capability to generate lymphatic invasion and nodal metastasis may have occurred from spontaneous genetic mutation without any impact from microenvironmental mechanisms.

As our understanding of metastatic disease continues to evolve and chemotherapeutic agents become more effective, it is certain that a diverse group of patients with chronically stable metastatic disease will emerge. The natural history of metastatic lesions will be of particular importance to this group and may significantly alter the course of their cancer therapy requirements. While this case raises several intriguing clinical and mechanistic questions for the oncologist, the therapeutic implications are uncertain and will require more specific clarification in the future.
